# Deciphering core proteins of osteoporosis with iron accumulation by proteomics in human bone

**DOI:** 10.3389/fendo.2022.961903

**Published:** 2022-10-14

**Authors:** Aifei Wang, Hui Zhang, Guangfei Li, Bin Chen, Junjie Li, Tao Zhang, Baoshan Liu, Zihou Cao, Gongwen Liu, Peng Jia, Youjia Xu

**Affiliations:** ^1^ Department of Orthopedics, Second Affiliated Hospital of Soochow University, Suzhou, China; ^2^ Cambridge-Suda Genomic Resource Centre, Soochow University, Suzhou, China; ^3^ Department of Orthopedics, Suzhou Hospital of Traditional Chinese Medicine, Suzhou, China; ^4^ Osteoporosis Institute, Soochow University, Suzhou, China

**Keywords:** osteoporosis, iron accumulation, proteomics, human bone, core proteins

## Abstract

Iron accumulation is an independent risk factor for postmenopausal osteoporosis, but mechanistic studies of this phenomenon are still focusing on molecular and genetic researches in model animal. Osteoporosis with iron accumulation is a distinct endocrine disease with complicated pathogenesis regulated by several proteins. However, the comprehensive proteome-wide analysis of human bone is lacking. Using multiplex quantitative tandem mass tag-based proteomics, we detected 2900 and quantified 1150 proteins from bone of 10 postmenopausal patients undergoing hip replacement. Comparing with non-osteoporosis patients, a total of 75 differentially expressed proteins were identified, comprising 53 downregulated proteins and 22 upregulated proteins. These proteins primarily affect oxidoreductase activity, GTPase activity, GTP binding, and neural nucleus development, were mainly enriched in neural, angiogenesis and energy-related pathways, and formed complex regulatory networks with strong interconnections. We ultimately identified 4 core proteins (GSTP1, LAMP2, COPB1, RAB5B) that were significantly differentially expressed in the bone of osteoporosis patients with iron accumulation, and validated the changed protein level in the serum of the medical examination population. Our systemic analysis uncovers molecular insights for revealing underlying mechanism and clinical therapeutics in osteoporosis with iron accumulation.

## Introduction

Osteoporosis is a systemic metabolic bone disorder characterized by low bone mass, deterioration of bone microstructure, and increased bone fracture risk among elderly (1, 2). Osteoporosis is the most common age-related bone disease worldwide, especially in postmenopausal women (3–5). With the rapid growth of an ageing population, the number of patients with osteoporosis is sharply increasing, and osteoporosis results in a substantial burden on both public health and economy (6). Ageing and oestrogen decline are two risk factors of the most common types of postmenopausal osteoporosis. However, recent studies have found a strong correlation between the incidence of postmenopausal osteoporosis and iron accumulation (7).

Iron, an abundant metallic element, plays an essential role in many human physiological processes, but excessive iron is harmful to multiple organs, including bones (8, 9). Thalassemia and sickle cell anaemia patients suffer from bone loss mainly due to iron accumulation, and the level of ferritin increases with age in osteoporosis of postmenopausal women (10, 11). Thus, iron accumulation is an independent risk factor for osteoporosis and can significantly accelerate the loss of bone mass in osteoporosis, especially in postmenopausal women (12, 13). In animal models, including mice, rats and zebrafish, iron accumulation triggers osteoporosis bone phenotype, but the underlying mechanism remains elusive (14, 15). Therefore, clarifying the mechanisms involved in osteoporosis with iron accumulation is urgently needed to shine a light on the effective clinical prevention and treatment of osteoporosis with iron accumulation in the future.

The Human Genome Project has been completed, life science research has gradually entered the post-genomic era charactered with functional genomics and proteomics (16, 17). Much research has been devoted to the transcriptional kinetics in osteoporosis based on transcriptome analysis, though several proteins modulate the occurrence and development of osteoporosis. Thus, increasing attention is now turning towards protein changes in osteoporosis. Currently, proteomic analyses to identify protein signature of osteoporosis have been performed on human serum and bone-related cell lines (18–22), but the proteomic analysis of osteoporosis with iron accumulation has not been conducted in human bone.

Changes in bone is the most direct manifestation of bone metabolism, it is very meaningful to directly understand the turnover in core proteins in the bone of osteoporosis patients with iron accumulation. We applied the TMT-based proteomics technique to integrally analyse differentially expressed proteins in bone tissue between the non-osteoporosis and osteoporosis with iron accumulation groups. Four core proteins of osteoporosis with iron accumulation are found. This study provides new ideas for further research on the mechanism of osteoporosis with iron accumulation and the discovery of therapeutic targets.

## Materials and methods

### Sample preparation

This research complies with the ethical regulations for work with human bone tissue samples. All participants were recruited from the Department of Orthopaedics, the Second Affiliated Hospital of Soochow University, China. We recruited patients who were diagnosed with one-sided femoral neck fracture, and needed to be treated with hip replacement surgery. Dual-energy X-ray absorptiometry (Hologic Delphi A; Hologic, Bedford, MA, USA) was applied to perform bone mineral density (BMD) examination on each patient 2–3 days before undergoing hip replacement surgery. Exclusion criteria included infection, tumour, developmental dysplasia of the hip, femoral head necrosis, osteomalacia, coagulopathy, renal insufficiency, hip surgery history, antiosteoporosis treatment history, and diseases that affect bone metabolism, such as thyroid disease, parathyroid disease, adrenal disease, and diabetes. A total of 18 patients were initially included in the study, 5 patients were excluded because they had received antiosteoporosis treatment, and 3 patients refused to sign informed consent, and finally 10 patients were included in the study. We distributed the patients into two groups according to their hip T-scores and serum ferritin: the non-osteoporosis (A) group was assigned T ≥ -1.2 and Fer ≤ 200nmol/mL, osteoporosis with iron accumulation (B) group was assigned T ≤ -3.2 and Fer ≥ 200nmol/mL.

An appropriate amount of femoral head tissue was obtained from a point 0.5 cm below the cartilage where the round ligament attaches to the femoral head, and all tissue samples were stored at -80°C. Frozen specimens were kept on dry ice and cut to ~50 mg of tissue from each sample to be used for proteomic analyses. Samples were first ground in liquid nitrogen, and then, the powder was transferred to a 5 mL centrifuge tube and sonicated three times on ice using a high-intensity ultrasonic processor (Scientz) in lysis buffer (including 1% Triton X-100, 10 mM dithiothreitol, 1% protease inhibitor cocktail, 50 μM PR-619, 3 μM TSA, 50 mM NAM and 2 mM EDTA). An equal volume of Tris-saturated phenol (pH 8.0) was added; then, the mixture was further vortexed for 5 min. After centrifugation (4°C, 10 min, 5 000 g), the upper phenol phase was transferred to a new centrifuge tube. Proteins were precipitated by addition of at least four volumes of ammonium sulfate-saturated methanol and incubated at -20°C for at least 6 h. After centrifugation at 4°C for 10 min, the supernatant was discarded. The remaining precipitate was washed with ice-cold methanol once, followed by three washes with ice-cold acetone. The protein was redissolved in 8 M urea, and the protein concentration was determined using a BCA kit according to the manufacturer’s instructions.

The work with tissue samples was approved under JD-LK-2020-027-01 by The Second Affiliated Hospital of Soochow University, and the work with serum samples was approved under SUDA20200424A04 by Soochow University.

### Trypsin digestion

For digestion, the protein solution was reduced with 5 mM dithiothreitol for 30 min at 56°C and alkylated with 11 mM iodoacetamide for 15 min at room temperature in the dark. The protein sample was then diluted by addition of 100 mM TEAB to urea concentrations less than 2 M. Finally, trypsin was added at a 1:50 trypsin-to-protein mass ratio for the first digestion overnight and a 1:100 trypsin-to-protein mass ratio for a second 4 h digestion.

### TMT labelling

After trypsin digestion, the peptides were desalted using a Strata X C18 SPE column (Phenomenex) and vacuum dried. Peptides were reconstituted in 0.5 M TEAB and processed using a TMT kit according to the manufacturer’s protocol. Briefly, one unit of TMT reagent was thawed and reconstituted in acetonitrile. The peptide mixtures were then incubated for 2 h at room temperature and pooled, desalted and dried by vacuum centrifugation.

### HPLC fractionation

The tryptic peptides were fractionated *via* high pH reverse-phase HPLC using an Agilent 300Extend C18 column (5 μm particles, 4.6 mm ID, 250 mm length). Briefly, peptides were first separated with a gradient of 8% to 32% acetonitrile (pH 9.0) over 60 min into 60 fractions. Then, the peptides were combined into 18 fractions and dried by vacuum centrifugation.

### LC–MS/MS analysis

The tryptic peptides were dissolved in 0.1% formic acid (solvent A) and directly loaded onto a homemade reversed-phase analytical column (15 cm length, 75 μm i.d.). The gradient comprised an increase from 6% to 23% solvent B (0.1% formic acid in 98% acetonitrile) over 26 min, 23% to 35% over 8 min and a climb to 80% over 3 min, holding at 80% for the last 3 min, all at a constant flow rate of 400 nL/min on an EASY-nLC 1000 UPLC system. The peptides were subjected to a nanospray ionization (NSI) source followed by tandem mass spectrometry (MS/MS) in a Q ExactiveTM Plus mass spectrometer (Thermo) coupled online to an ultra-performance liquid chromatography (UPLC) instrument. The electrospray voltage applied was 2.0 kV. The m/z scan range was 350 to 1800 for a full scan, and intact peptides were detected in the Orbitrap at a resolution of 70,000. Peptides were then selected for MS/MS using a normalized collision energy (NCE) setting of 28, and the fragments were detected in the Orbitrap at a resolution of 17,500. A data-dependent procedure that alternated between one MS scan followed by 20 MS/MS scans with 15.0 s dynamic exclusion was conducted. Automatic gain control (AGC) was set at 5E4. The fixed first mass was set at 100 m/z.

### Database search

The resulting MS/MS data were processed using the MaxQuant search engine (v.1.5.2.8). Tandem mass spectra were searched against the database concatenated with the reverse decoy database. Trypsin/P was specified as a cleavage enzyme, allowing up to 2 missing cleavages. The mass tolerance for precursor ions was set as 20 ppm in the first search and 5 ppm in the main search, and the mass tolerance for fragment ions was set as 0.02 Da. Carbamidomethyl on Cys was specified as a fixed modification, and oxidation of Met was specified as a variable modification. The false discovery rate (FDR) was adjusted to < 1%, and the minimum score for peptides was set at > 40.

### Principal component analysis (PCA)

Principal component analysis of 10 bone tissue samples and proteome were performed using SIMCA software (version 14.0; Umetrics, Umea, Sweden).

### Enrichment of gene ontology analysis

Proteins were classified by GO annotation into three categories: biological process, cellular compartment and molecular function. For each category, a two-tailed Fisher’s exact test was employed to test the enrichment of the differentially expressed protein against all identified proteins. GO terms with a corrected p value < 0.05 were considered significant.

### Enrichment of pathway analysis

The Encyclopedia of Genes and Genomes (KEGG) Pathway, WikiPathways, Hallmark Gene Sets and Reactome Gene Sets database were used to identify enriched pathways using a two-tailed Fisher’s exact test to test the enrichment of the differentially expressed proteins against all identified proteins. Pathways with a corrected p value < 0.05 were considered significant.

### Membership analysis

The membership analysis of differently expressed proteins on bone and iron metabolism were conducted on the bioinformation website Metascape (23).

### Protein–protein interaction network

All differentially expressed protein database accessions or sequences were searched against the STRING database version 10.5 for protein–protein interactions. Only interactions between the proteins belonging to the searched data set were selected, thereby excluding external candidates. STRING defines a metric called the “confidence score” to define interaction confidence; we fetched all interactions that had a confidence score >0.7 (high confidence). The interaction network from STRING was visualized Cytoscape, and ClueGO was used to visualize the nonredundant biological terms for large clusters of genes in a functionally grouped network.

### ELISA assays

The levels of GSTP1, LAMP2, COPB1 and RAB5B in serum were detected using the following ELISA kits: GSTP1 ELISA Kit (RayBio, # ELH-GSTP1, US); LAMP2 ELISA Kit (Abcam, #ab277449, UK); COPB1 ELISA Kit (Abbexa, #ABX507968, US); RAB5B ELISA Kit (SAB, #EK7817, US).

### Statistical analysis

The quantitative data are expressed as the mean ± standard deviation (SD). Correlation analysis is using Pearson’s correlation analysis, Student’s t test and one-way ANOVA followed by Bonferroni *post hoc* tests were used for the comparison analysis using SPSS 21.0 software. *P*<0.05 or 0.01 was considered statistically significant.

## Results

### Weak correlation between classical markers of bone metabolism and bone density in iron accumulation with osteoporosis

To character the protein signature of osteoporosis with iron accumulation, we collected serum and bone tissue from10 postmenopausal women who underwent hip replacement surgery due to hip fracture, 5 were non-osteoporotic with normal serum ferritin (Group NOP), and 5 were osteoporotic patients with iron accumulation (Group OIA). The mean age at baseline was 80.2 ± 7.7 years, the mean BMI was 21.53 ± 4.27 kg/cm^2^, and the mean T scores of the hips, serum ferritin, PINP and β-CTX are shown in **Table 1**.

**Table 1:** Baseline characteristics of the patients in our cohort.

Characteristics	NOP (n=5) Mean (95% CI)	OIA (n=5) Mean (95% CI)	*P*
Age (years)	79.2 ± 9.28	81.2 ± 7.73	0.721
Height (cm)	155.6 ± 3.78	154 ± 4.69	0.569
Weight (kg)	57.6 ± 12.18	45.8 ± 8.56	0.114
BMI (kg/cm2)	23.71 ± 4.39	19.34 ± 3.78	0.130
T score			
Hip	0.06 ± 1.30	-3.64 ± 0.38	< 0.001
Lumbar	-1.22 ± 1.16	-3.48 ± 1.08	0.013
BMD			
Hip	0.91 ± 0.09	0.51 ± 0.06	< 0.001
Lumbar	0.96 ± 0.14	0.68 ± 0.10	0.008
Femoral neck	0.94 ± 0.15	0.49 ± 0.05	< 0.001
Greater trochanter	0.84 ± 0.35	0.39 ± 0.35	0.021
Fer (nmol/mL)	106.38 ± 26.07	332.24 ± 65.41	0.004
PINP (ng/mL)	81.17 ± 58.14	83.03 ± 42.95	0.956
β-CTX (ng/mL)	536.72 ± 132.80	576.16 ± 389.85	0.836

The study samples are all postmenopausal patients. BMI, Body Mass Index; BMD, Bone Mineral Density; Fer, Ferritin; PINP, procollagen type I N-propeptide; β-CTX, β-isomerized C-terminal telopeptides.

**Figure 1:**
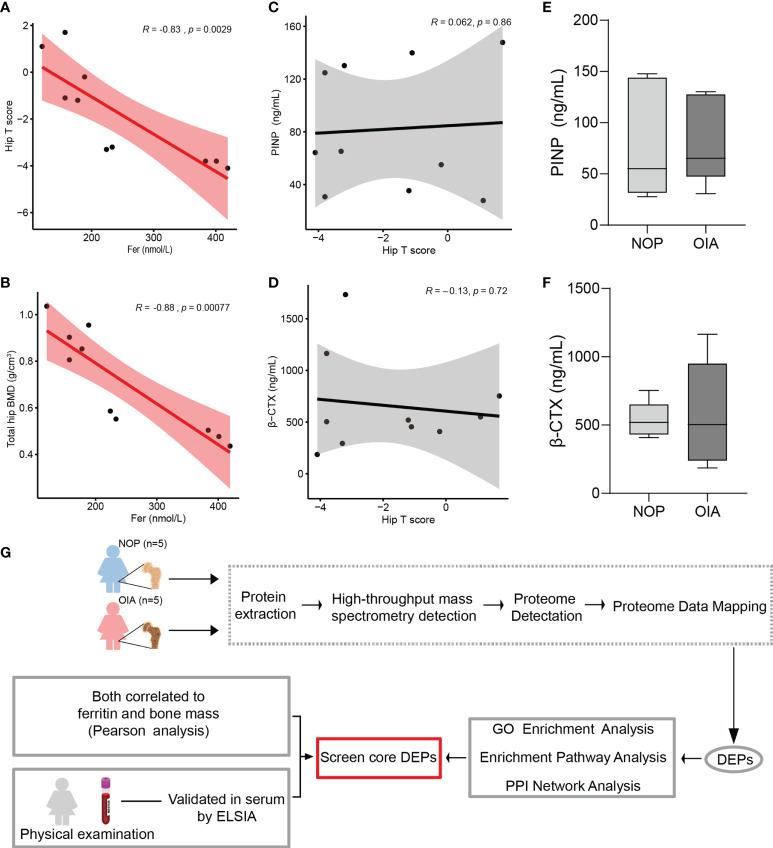
Correlation analysis of clinical index, proteomic analysis workflow. **(A)** Pearson correlation analysis of hip T score and serum ferritin. **(B)** Pearson correlation analysis of total hip bone mineral density and serum ferritin. **(C)** Pearson correlation analysis of serum PINP and hip T score. **(D)** Pearson correlation analysis of serum β-CTX and hip T score. **(E)** Serum PINP level of patients from Group NOP and OIA. **(F)** Serum β-CTX level of patients from Group NOP and OIA. **(G)** Workflow for quantitative mass spectrometry profiling.

Serum ferritin is known as a marker of iron accumulation. Thus, a Pearson correlation analysis was used to test the correlation between serum ferritin and bone mass. We found a strong correlation between serum ferritin and bone mass indices, including hip T score, total hip BMD, lumbar T score, lumbar BMD, femoral neck BMD, and greater trochanter BMD (**Figures 1A, B** and **Supplementary Figures 1A-D**). This result indicates that iron accumulation is closely related to postmenopausal osteoporosis. Moreover, age, height, weight, and BMI did not show a correlation with serum ferritin, and serum PINP and β-CTX were also not associated with serum ferritin (**Supplemental Figures 2A-F**). Unexpectedly, the levels of serum PINP and β-CTX, known bone metabolic biomarkers, did not show a correlation with the hip T score and did not differ significantly between the groups (**Figures 1C–F**). These results suggest that traditional clinical serum markers may not intuitively reflect the bone density in osteoporosis with iron accumulation, and its core proteins which tightly correlated with iron urgently need to be discovered.

### Identification of differentially expressed proteins in the bone of osteoporosis patients with iron accumulation

To identify the changed proteins of osteoporosis patients with iron accumulation, we collected bone from the femoral heads of postmenopausal women who underwent hip replacement surgery, and proteome of these samples were analysed *via* high-resolution mass spectrometry (**Figure 1G** and **Supplemental Figure 3**). The peptide lengths and mass error distribution were used to access each identified peptide. The distribution of the mass error was less than 5 ppm and the lengths of peptides varied from 7 to 20 amino acids, suggesting that the sample preparation met the standard (**Supplement Figures 4A, B** **)**. In total, our proteomic profile assessment of the 10 bone tissue samples led to the discovery of 2900 proteins, of which 1150 were specifically quantified.

**Figure 2:**
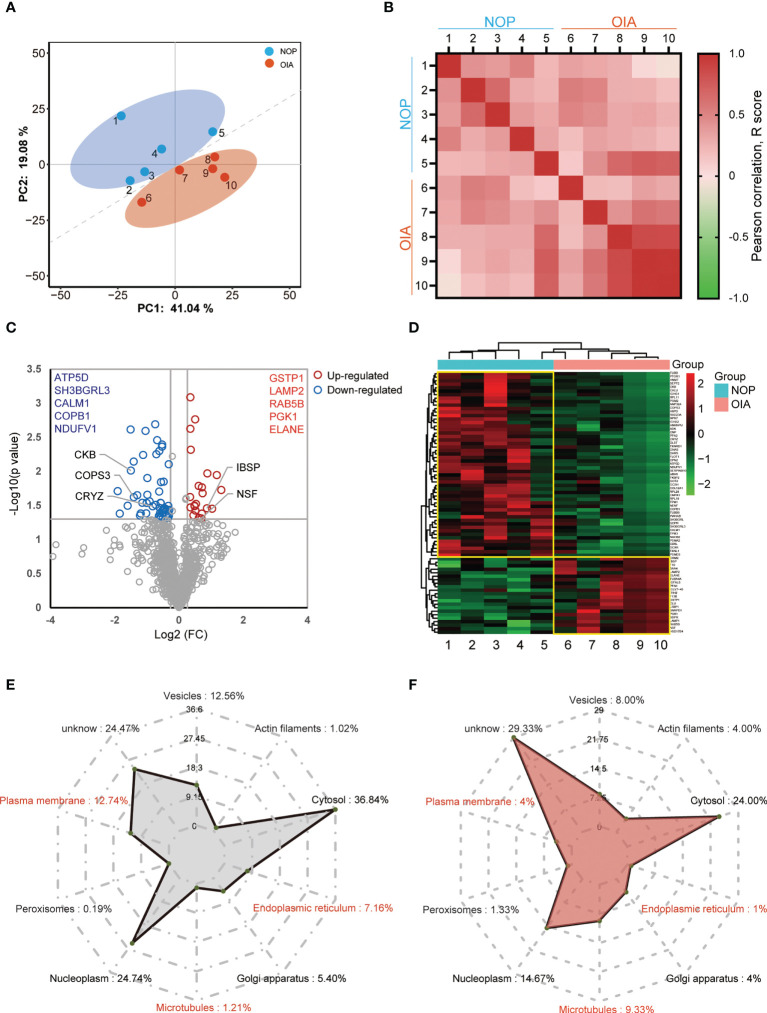
Quantitative proteomics analysis of human bone from osteoporosis patients with iron accumulation. **(A)** Principal component analysis (PCA) plot showing unsupervised clustering among the 10 samples, demonstrating clear distinction between two Groups. **(B)** Pearson correlation analysis of proteomic data from 10 samples. **(C)** Volcano plots comparing bone proteome of Group NOP and OIA. **(D)** Heatmap and cluster of the differentially expressed proteins. **(E, F)** Subcellular localization chart of NDEPs **(E)** and DEPs **(F)**. The amount of protein in each section is expressed as a percentage.

Replicate proteome measurements showed high consistency and independently recapitulate the difference between NOP and OIA (**Figure 2A**). High intra-group consistency within each group was observed, especially in OIA, and the synchronous protein expression is disappeared on inter-group correlation analysis (**Figure 2B**). A volcano plot was used to display the proteomic reprogramming (**Figure 2C**). Of a total of 1,150 quantified proteins, 22 proteins were upregulated (**Figure 2C**, red circle), whereas 53 proteins were depleted in OIA (**Figure 2C**, blue circle). Substantial but not the strongest changes in 5 known molecules that related to eBMD were observed in the volcano plot, demonstrating reliability of these data and other potential core proteins, and the 5 most upregulated and 5 most profoundly downregulated proteins are shown. Of the 75 differentially expressed proteins, 66 (88%) proteins were expressed in murine osteoblasts, and 54 (72%) were expressed in murine osteoclasts (**Supplement Table 1**). The heatmap of the differentially expressed proteins (DEPs) is shown in **Figure 2D**, which indicated that the expression of these proteins changed obviously in the bone tissue of osteoporosis patients with iron accumulation. A subcellular localization analysis based on Protein Atlas showed that both no- differentially expressed proteins (NDEPs) and DEPs mainly locate in cytosol and nucleoplasm, but DEPs is more positioned in microtubules, less positioned in plasma membrane and endoplasmic reticulum (**Figures 2E, F**). Protein types of NDEPs and DEPs are similar (**Supplement Figure 5**). These results suggest that this human bone proteomic data is stable and the core proteins can be screened from 75 DEPs.

### GO terms and pathway enrichment analysis for DEPs

**Figure 3:**
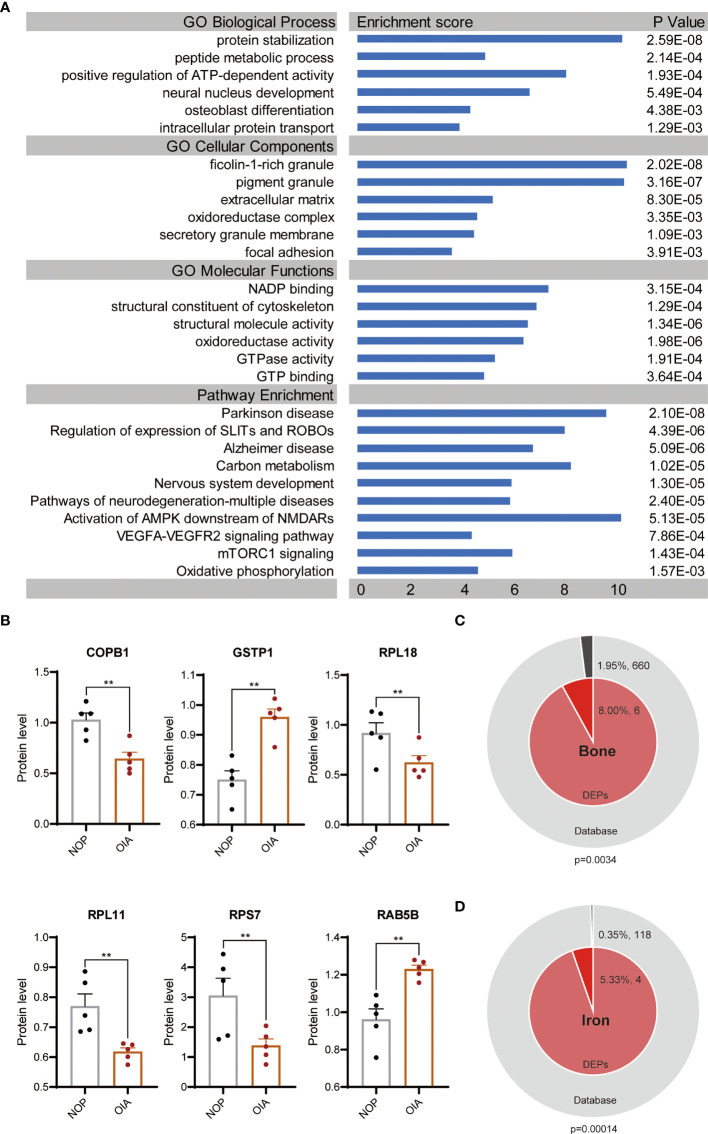
GO terms and pathway enrichment analyses of DEPs between OIA and NOP samples. **(A)** GO and pathway analysis of the 75 DEPS. **(B)** Changes in 6 top enriched proteins of GO terms in the osteoporosis with iron accumulation. **(C)** Membership analysis of DEPs associated with bone metabolism. **(D)** Membership analysis of DEPs associated with iron metabolism. **P<0.01.

To further elaborate the function of differentially expressed proteins, we performed GO enrichment and pathway enrichment analysis (**Figure 3A**). These proteins were also enriched in a wide range of biological processes, such as positive regulation of ATP-dependent activity, neural nucleus development and osteoblast differentiation. The top enriched CC terms were ficolin-1-rich granule, extracellular matrix, and focal adhesion. The DEPs were significantly enriched in MF terms related to redox and energy metabolism.

We performed enrichment pathway analysis with 4 databases, KEGG Pathway, WikiPathways, Hallmark Gene Sets and Reactome Gene Sets. These pathways were neurodegeneration-multiple diseases, followed by Parkinson’s disease, Alzheimer’s disease. Other than that, carbon metabolism, oxidative phosphorylation, VEGFA-VEGFR2 signalling pathway, mTORC1 signalling, SLITs and ROBOs and nervous system development were highly enriched (**Figure 3A**).

Six proteins, COPB1, GSTP1, RPL18, RPL11, RPS7 and RAB5B, were hit in results of all three enriched GO terms pertaining BP, CC and MF. The significant changes of these proteins in osteoporosis with iron accumulation were validated in primary proteomic data (**Figure 3B**). To test the association between differentially expressed proteins and bone metabolism and iron metabolism, we conducted a membership analysis for these proteins in bone-related terms/pathways (GO:0001649, GO:0001503, hsa04935, hsa04915) and iron-related terms/pathways (GO:0035732, GO:0051539, hsa04971). As expected, the percentage of bone-related proteins among the DEPs was significantly high, and 5.33% of the DEPs were related to iron metabolism, which was higher than the proportion of iron-related proteins in all protein databases (**Figures 3C, D**).

Together, above analyses indicated that DEPs were mainly correlated with energy metabolism, nervous system disease, mTORC1, SLIT and ROBO pathways. Comparing with total proteins, DEPs were more significant in bone and iron related terms.

### PPI network and pathway cluster analysis of DEPs

**Figure 4:**
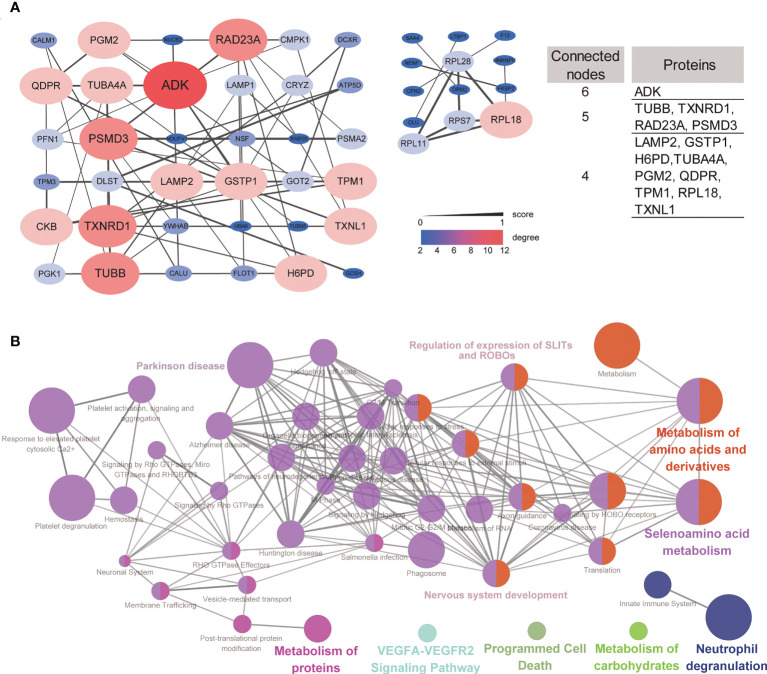
Protein–protein network and network of terms/pathways. **(A)** Protein–protein interaction networks of up- and downregulated signatures, and DEPs with connected nodes >3 were listed. **(B)** Network of terms/pathways identified using ClueGO highlighting the terms/pathways of differentially expressed proteins.

To understand the potential interactions between differentially expressed proteins, a PPI network was constructed. Based on STRING predictions, we found that these differentially expressed proteins had multiple interactions and two clusters in the network were discovered, and these clusters were mainly associated with proteasome and nervous disease. Construction of two PPI networks revealed 49 nodes and 65 edges. 14 DEPs, such as ADK, PSMD3, LAMP2 and GSTP1 have higher degrees than the other nodes in the PPI network suggesting these are hub proteins in DEPs (**Figure 4A**).

ClueGO can visualize the nonredundant biological terms for large clusters of genes in a functionally grouped network. Here, we generated a network of terms/pathways to gain a better understanding of these differentially expressed proteins. The differentially expressed proteins were mainly involved in nervous system development, Parkinson’s disease, regulation of the expression of SLITs and ROBOs, and metabolism of amino acids and derivatives (**Figure 4B**). These results were consistent with the GO and pathway enrichment analyses.

### Core proteins of osteoporosis with iron accumulation

**Figure 5:**
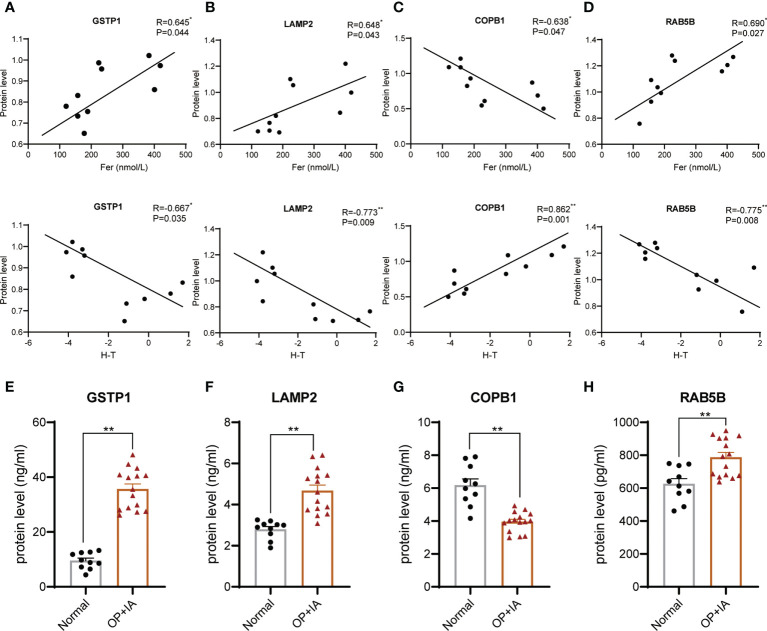
Core proteins were screened from the DEPs and validated in serum. **(A-D)** Correlation analysis of hip T score, serum ferritin and protein levels of core proteins, GSTP1 **(A)**, LAMP2 **(B)**, COPB1 **(C)**, and RAB5B **(D)**. (Pearson correlation analysis). **(E-H)** Serum protein levels of physical exam volunteers were tested by ELSIA kits. *P<0.05, **P<0.01.

The enrichment and PPI network analysis indicate several important proteins in osteoporosis with iron accumulation. Then, we attempt to further screen out core proteins. By analysing the primary proteomic data of DEPs, we found that most of the DEPs were related to bone mass but only 4 DEPs were related to both bone mass and serum ferritin (**Figures 5A–D**, **Table 2** and **Supplement Tables 2, 3**). Therefore, we speculate that these 4 DEPs (GSTP1, LAMP2, COPB1, RAB5B) may be novel candidate core proteins of osteoporosis with iron accumulation. Furthermore 25 samples of serum from postmenopausal women undergoing physical examination were collected for validation by ELSIA, including 10 normal and 15 osteoporosis with iron accumulation serum samples, as expected, these protein changes in bone tissue were also confirmed in serum samples (**Figures 5E–H**). Moreover, these core proteins were either hits in highly enriched GO terms or hubs in PPI network (**Figures 3B** and **4A**). Together, these 4 core proteins (GSTP1, LAMP2, COPB1, and RAB5B) may account for osteoporosis with iron accumulation, and potentially be a serum marker for it.

**Table 2:** Proteins differentially abundant in osteoporosis and iron accumulation.

		Associations with bone mass		Associations with serum ferritin	
Protein symbol	Protein name	R value	*P* value	R value	*P* value
GSTP1	Glutathione S-transferase P	-0.667*	0.035	0.654*	0.044
LAMP2	Lysosome-associated membrane glycoprotein 2	-0.773**	0.009	0.648*	0.043
COPB1	Coatomer subunit beta	0.862**	0.001	-0.638*	0.047
RAB5B	Ras-related protein Rab-5B	-0.775**	0.008	0.690*	0.027

*p<0.05, **p<0.01.

## Discussion

Our knowledge of the protein changes in osteoporosis with iron accumulation is lacking, and the assessment of holistic protein expression under pathological conditions is mainly limited to serum or blood cells. Using a TMT-based high-throughput proteomics strategy to directly screen differentially abundant proteins in the bone tissue of osteoporosis patients with iron accumulation, we detected 2900 proteins in the bone of the patient cohort, and 1150 proteins could be quantitatively analysed in each single sample. Using the 1.2-fold change and *P* value <0.05 cutoff, we found that 22 proteins in the bone of the patients with osteoporosis with iron accumulation were increased compared with those of the NOP group, and 53 were decreased. Multiple bioinformatics tools were used to analyse these proteins. We further confirmed the relationship of these proteins to iron metabolism and bone metabolism. Several differentially expressed proteins showed a significant correlation with both serum ferritin and bone mass.

Changes in the proteins in this study suggest that these differentially expressed proteins may play essential roles in the pathogenesis of osteoporosis with iron accumulation. The results of GO enrichment analysis showed that differentially expressed proteins were related to redox, energy metabolism and neural processes. Energy metabolism plays a role in bone metabolism. Research has shown that glycolysis is the major metabolic pathway to meet the demand for ATP during osteoblast differentiation, and osteoblast dysfunction in clinical diseases, including diabetes, anorexia nervosa, and ageing, results from impaired substrate availability, ultimately leading to skeletal fragility and osteoporotic fractures (24). Bone resorption is a process that consumes large amounts of adenosine triphosphate (ATP) produced by glycolysis and oxidative phosphorylation, osteoclasts are the main performers of this biological process, and glucose, fatty acids and amino acids can also be used as substrates to produce energy through oxidative phosphorylation according to recent research (25). Iron is essential for cell energy metabolism, and iron can positively affect the activity of mitochondrial aconitase and increase mitochondrial oxygen consumption and ATP formation *via* oxidative phosphorylation (26). Therefore, it makes sense that the differentially expressed proteins are related to energy metabolism. The brain is the largest energy-consuming organ in humans, and abnormal iron metabolism impairs energy metabolism in a brain region-specific manner, particularly in hippocampal neurons (27). Similar to the results of GO enrichment analysis, pathway enrichment analysis for differentially expressed proteins also suggests a relationship with neurological diseases, and there is mounting evidence that neurological conditions are associated with a significantly increased risk of osteoporosis and fractures. Parkinson’s disease was identified in the Global Longitudinal Study of Osteoporosis in Women study as significantly associated with osteoporosis (28). These studies show a strong link between iron, osteoporosis and the nervous system, but the mechanism still needs to be further explored.

Pathway enrichment results based on 4 databases in addition to the above related Alzheimer’s disease and oxidative phosphorylation also included the VEGFA-VEGFR2 signalling pathway, mTORC1 signalling and regulation of the expression of SLITs and ROBOs. VEGFA is an important proangiogenic factor, and the generation of blood vessels is essential to maintain bone homeostasis (29). Abundant bone vessels can accelerate bone regeneration and healing of bone fractures (30). Therefore, the angiogenesis pathway may play an important role in osteoporosis with iron accumulation. Interestingly, consistent with our previous findings that iron accumulation impairs the osteogenesis and angiogenesis of osteoporosis *via* the osteoblastic mTORC1 pathway (31), these differentially expressed proteins of bone from osteoporosis patients with iron accumulation were also enriched in the mTORC1 pathway. SLITs and ROBOs mainly regulate nerve growth guidance and multisystem angiogenesis (32). Xu et al. found that *Slit3* knockout mice had reduced bone mass, mainly due to a decreased number of H-type blood vessels in the bone (33). Based on the network analysis, a majority of proteins were involved in proteasome and nervous disease, and proteins related to the proteasome were closely associated with post-transcriptional, translational, and post-translational regulation (34). These pathways may differ from the canonical bone metabolic pathway, and these functional pathways in osteoporosis with iron accumulation need to be further investigated.

**Figure 6:**
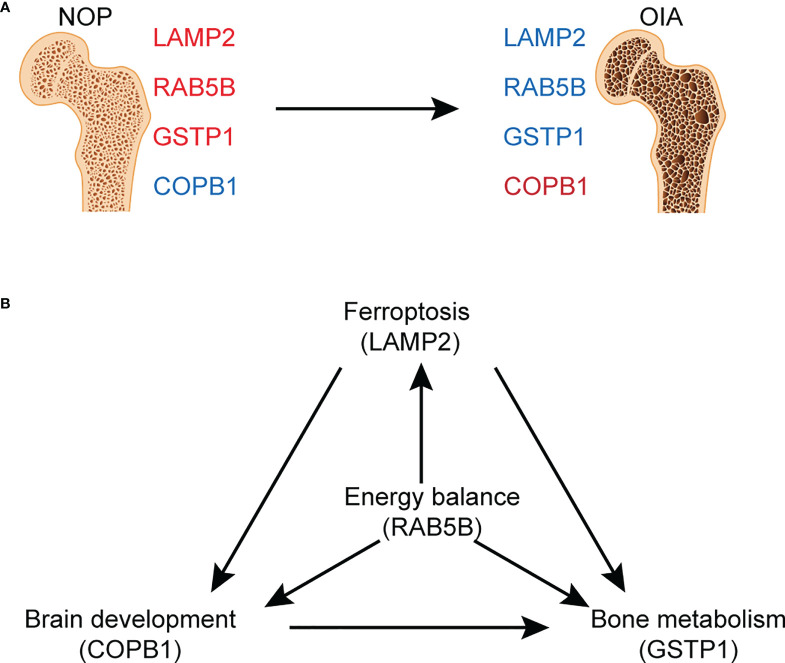
Graphed summary of four core proteins. **(A)** The changes of four core proteins between NOA and OIA. **(B)** The possible effects of four core proteins on the related pathogenesis.

Membership analysis of differentially expressed proteins demonstrated a strong correlation with bone metabolism and iron metabolism. Analysing the expression level of differentially expressed proteins with serum ferritin and bone mass by Pearson correlation analysis, we found that most of the differentially expressed proteins were related to either bone mass or serum ferritin. Proteins related to both ferritin and bone mass are the most critical proteins in osteoporosis with iron accumulation. Finally, we screened four candidate proteins of osteoporosis with iron accumulation, GSTP1, LAMP2, COPB1, and RAB5B (**Figure 6A**). Glutathione-S transferases (GSTs) are a family of enzymes involved in catalysing the detoxification of endogenous and exogenous substances by their conjugation with glutathione (GSH). GSTP1 belongs to the pi class of these enzymes, and iron can induce intracellular GSH/GST antioxidant system changes. Mlakar et al. found that glutathione S-transferases play a role in the bone remodelling process in an analysis of the GSTP1 genotypes and haplotype interactions in Slovenian post/premenopausal women (35). The protein encoded by LAMP2 is a member of a family of membrane glycoproteins. This glycoprotein provides selectins with carbohydrate ligands, and mutations of LAMP2 cause the classic triad of myopathy, cardiomyopathy and encephalopathy of Danon disease (DD) (36). LAMP2 deficiency reduces the cytosolic cysteine concentration, resulting in low glutathione (GSH), poor antioxidant capacity and mitochondrial lipid peroxidation, ultimately leading to ferroptosis (37). The relationship between LAMP2 and osteoporosis has not been examined. COPI coat complex subunit beta 1 (COPB1) is a protein subunit of the coatomer complex associated with nonclathrin-coated vesicles. The coatomer complex, also known as coat protein complex 1, forms in the cytoplasm and is recruited to the Golgi by activated guanosine triphosphatases (38). Depletion of COPI in cancer cells resulted in decreased cell survival and impaired autophagy and ER stress (39). A genetic study showed that COPB1 subunits are essential for brain development and human health (40). RAB5B is a member of the RAB subfamily of small GTPases and plays a role in cell migration and proliferation. LRRK2 kinase activity functions as a Rab5b GTPase activating protein and has been identified as a causative gene for Parkinson’s disease (41). However, no research on COPB1 or RAB5B has been focused on bone metabolism-related diseases. Based on this discussion, we speculate that these critical proteins are involved in the occurrence of osteoporosis with iron accumulation by affecting the interaction of ferroptosis, energy metabolism, brain development and bone metabolism. (**Figure 6B**) These proteins may provide new ideas for in-depth mechanistic studies, helping to discover novel therapeutic targets for osteoporosis with iron accumulation.

There are still some limitations in this study. Due to difficulty of obtaining clinical bone tissue samples, especially in people with iron accumulation, our sample size is limited. Osteoporosis with iron only occurs in a subset of the population, these core proteins may not account for other types of osteoporosis. In addition, we validated the core proteins level in serum of postmenopausal women undergoing physical examination, but not the bone tissue directly, and the specific functions and mechanisms of these proteins in bone metabolism need further research. Bone mineral density and bone strength are important indicators for evaluating bone quality, and bone mineral density is the most used clinical bone health index, because bone mineral density can be detected by low-radiation non-invasive DXA (Dual-emission X-ray Absorptiometry). Bone strength is determined by its material composition and structure. However, the most effective detection method of bone strength is bone biomechanics, which requires isolated bone samples for detection, so there is no standard detection method and bone strength parameters in clinical practice. In this proteomics study, we mainly used the index of bone mineral density, and further analysis of bone strength index may make our results more objective and comprehensive.

Iron accumulation is an independent risk factor for postmenopausal osteoporosis. Current postmenopausal osteoporosis proteomics research mainly focuses on serum proteomics research. Serum proteomics is often more advantageous in the discovery of biomarkers. However, changes in bone tissue make the most direct manifestation of osteoporosis. Therefore, bone tissue proteomics may be able to find more critical molecules than serum proteomics, but it is very difficult to obtain human bone tissue compared to serum. In addition, we also tested the screened core proteins serum level of 30 patients by ELISA. On the one hand, it is for preliminary verification, and on the other hand, it is to explore their application potential in serological detection.

To our knowledge, this is the first study to use a proteomics approach for human bone to explore the proteins of osteoporosis with iron accumulation, and we emphasize that the current study is a preliminary exploration of the proteins in bone tissue of osteoporosis patients with iron accumulation. By bioinformatic analysis and experimental validation, we identified 4 core proteins in osteoporosis with iron accumulation. We will focus on some potential target proteins in the future for in-depth research on the underlying mechanisms of osteoporosis with iron accumulation. Applications of proteomics in a larger population may be an effective means of discovering new biomarkers and useful in revealing the biological underpinnings of osteoporosis with iron accumulation.

## Data availability statement

The original contributions presented in the study are included in the article/**Supplementary Material**. Further inquiries can be directed to the corresponding authors.

## Ethics statement

The studies involving human participants were reviewed and approved by Ethics Committee for Second Affiliated Hospital of Soochow University. The patients/participants provided their written informed consent to participate in this study.

## Author contributions

Sample collection: AW, BC, HZ, and GWL; Data analysis: AW, BC, HZ, and TZ; Visualization: AW, GFL, and PJ; Supervision: HZ, BL, JL, and ZC; Overall project design: YX and AW; Writing—review and editing: AW, TZ, and YX. All authors contributed to the article and approved the submitted version.

## Funding

This work was supported by grants from National Key R&D Program of China (Grant No. SQ2021YFC2501702 to YJX), National Natural Science Foundation of China (Grant Nos. 82072474 to YJX, 81903326 to BC), Clinical Medicine Technology Project of Jiangsu Province (Grant No. BE2019661 to YJX), Health leading talents of Gusu City (Grant No. GSWS2019004 to YJX) and Postgraduate Research & Practice Innovation Program of Jiangsu Province (Grant No. KYCX20_2677 to AFW)

## Acknowledgments

We thank Yan Gao, Yuan Li, Zhipeng Liu, and Peng Zhang for collecting the human bone samples and PTM BIO for technical assistance with bone tissue proteomics.

## Conflict of interest

The authors declare that the research was conducted in the absence of any commercial or financial relationships that could be construed as a potential conflict of interest.

## Publisher’s note

All claims expressed in this article are solely those of the authors and do not necessarily represent those of their affiliated organizations, or those of the publisher, the editors and the reviewers. Any product that may be evaluated in this article, or claim that may be made by its manufacturer, is not guaranteed or endorsed by the publisher.
