# Supplementary material for: Deciphering core proteins of osteoporosis with iron accumulation by proteomics in human bone
Source: Front Endocrinol (Lausanne). 2022 Oct 14;13:961903. doi: 10.3389/fendo.2022.961903 (PMC9614156; doi:10.3389/fendo.2022.961903)
Supplement: Supplementary file 1 [file DataSheet_1.pdf]

Supplementary Materials for

Deciphering Core Proteins of Osteoporosis with Iron Accumulation by Proteomics in Human Bone

Aifei Wang[1 †], Hui Zhang[1 †], Guangfei Li[1†], Bin Chen[1 †], Junjie Li[1], Tao Zhang[4], Baoshan Liu [1], Zihou Cao[1], Gongwen Liu[2], Peng Jia[1]* and Youjia Xu[1,3]*

[1]Department of Orthopedics, Second Affiliated Hospital of Soochow University, Suzhou, China, [2]Department of Orthopedics, Suzhou Hospital of Traditional Chinese Medicine, Suzhou, China, [3]Osteoporosis Institute, Soochow University, Suzhou, China, [4]Cambridge-Suda Genomic Resource Centre, Soochow University, Suzhou, China

*Correspondence: Peng Jia, jiapengorthop@163.com
Youjia Xu, xuyoujia@suda.edu.cn
†These authors contributed equally to this work and share first authorship.

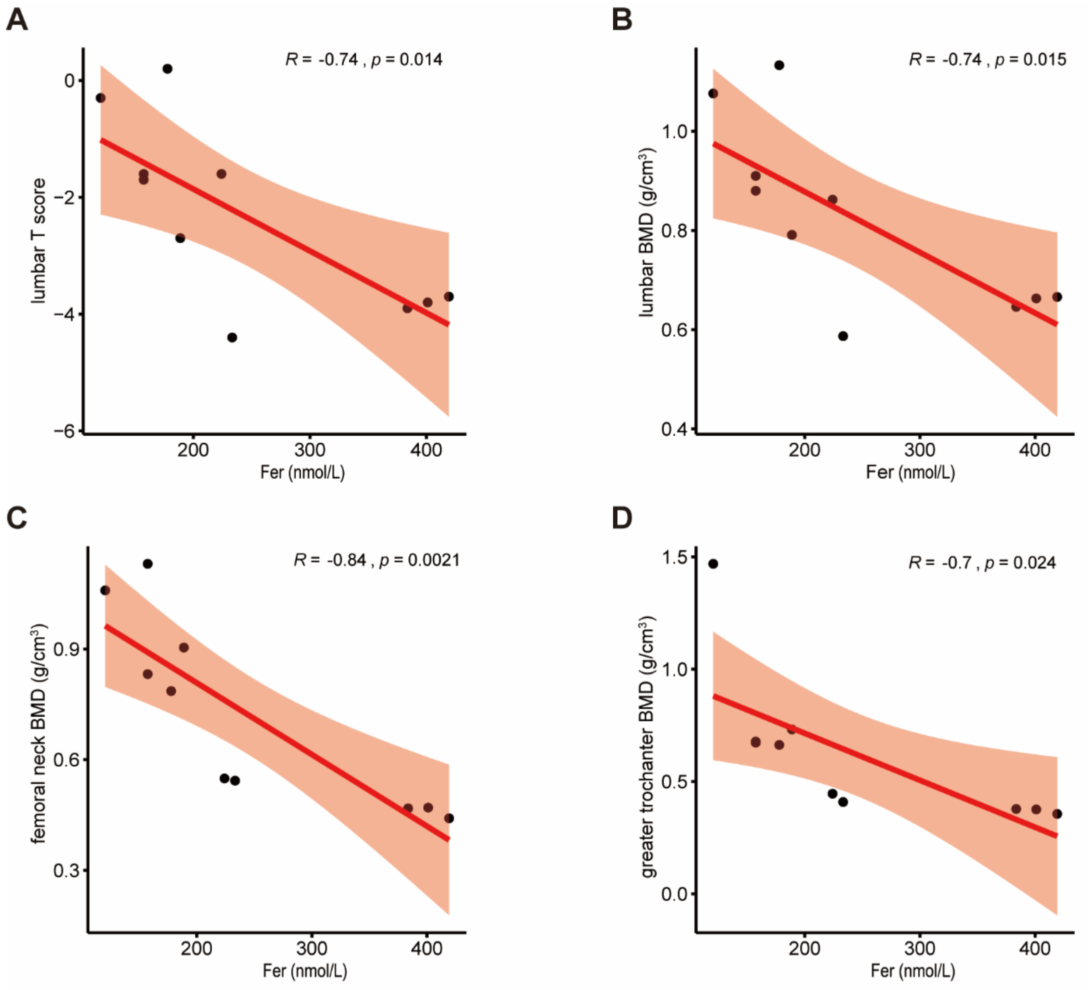

Supplementary Figure 1 | Correlation analysis of bone mass index and serum ferritin. **(A)** Pearson correlation analysis of lumbar T score and serum ferritin. **(B)** Pearson correlation analysis of lumbar BMD and serum ferritin. **(C)** Pearson correlation analysis of femoral neck BMD and serum ferritin. **(D)** Pearson correlation analysis of greater trochanter BMD and serum ferritin.

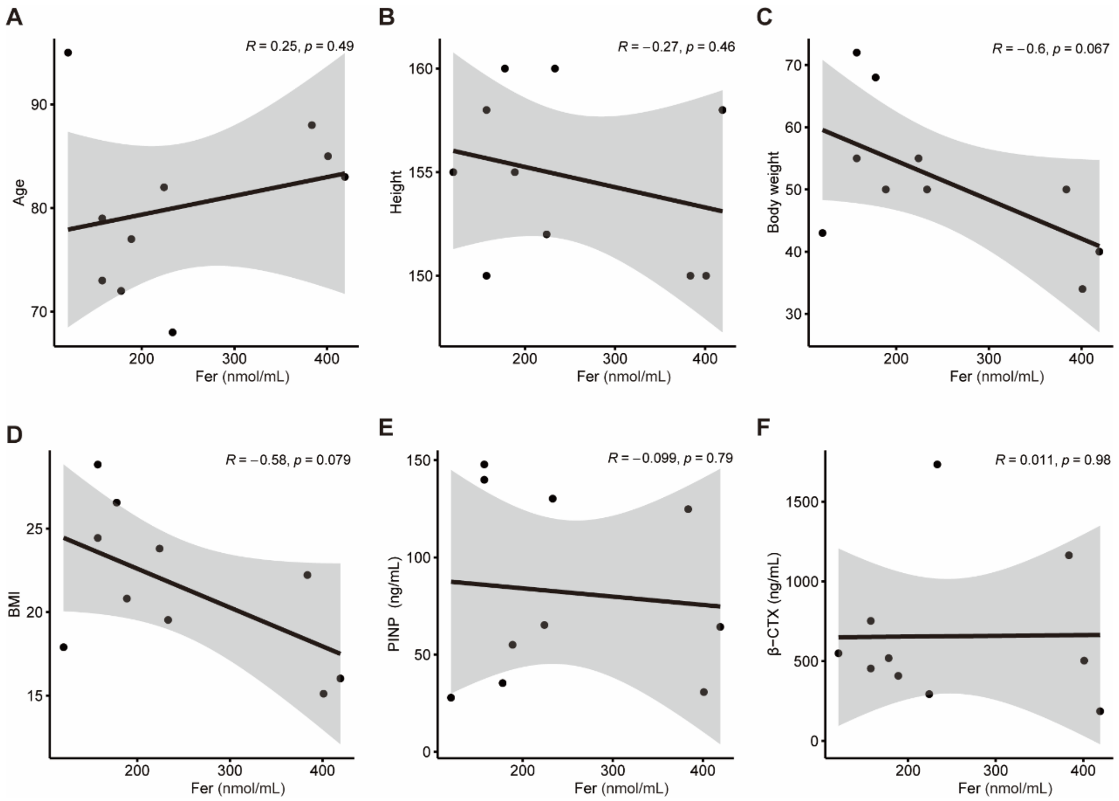

Supplementary Figure 2 | Correlation analysis of general clinical index and serum ferritin. **(A)** Pearson correlation analysis of age and serum ferritin. **(B)** Pearson correlation analysis of height and serum ferritin. **(C)** Pearson correlation analysis of body weight and serum ferritin. **(D)** Pearson correlation analysis of BMI and serum ferritin. **(E)** Pearson correlation analysis of serum PINP and serum ferritin. **(F)** Pearson correlation analysis of serum β-CTX and serum ferritin.

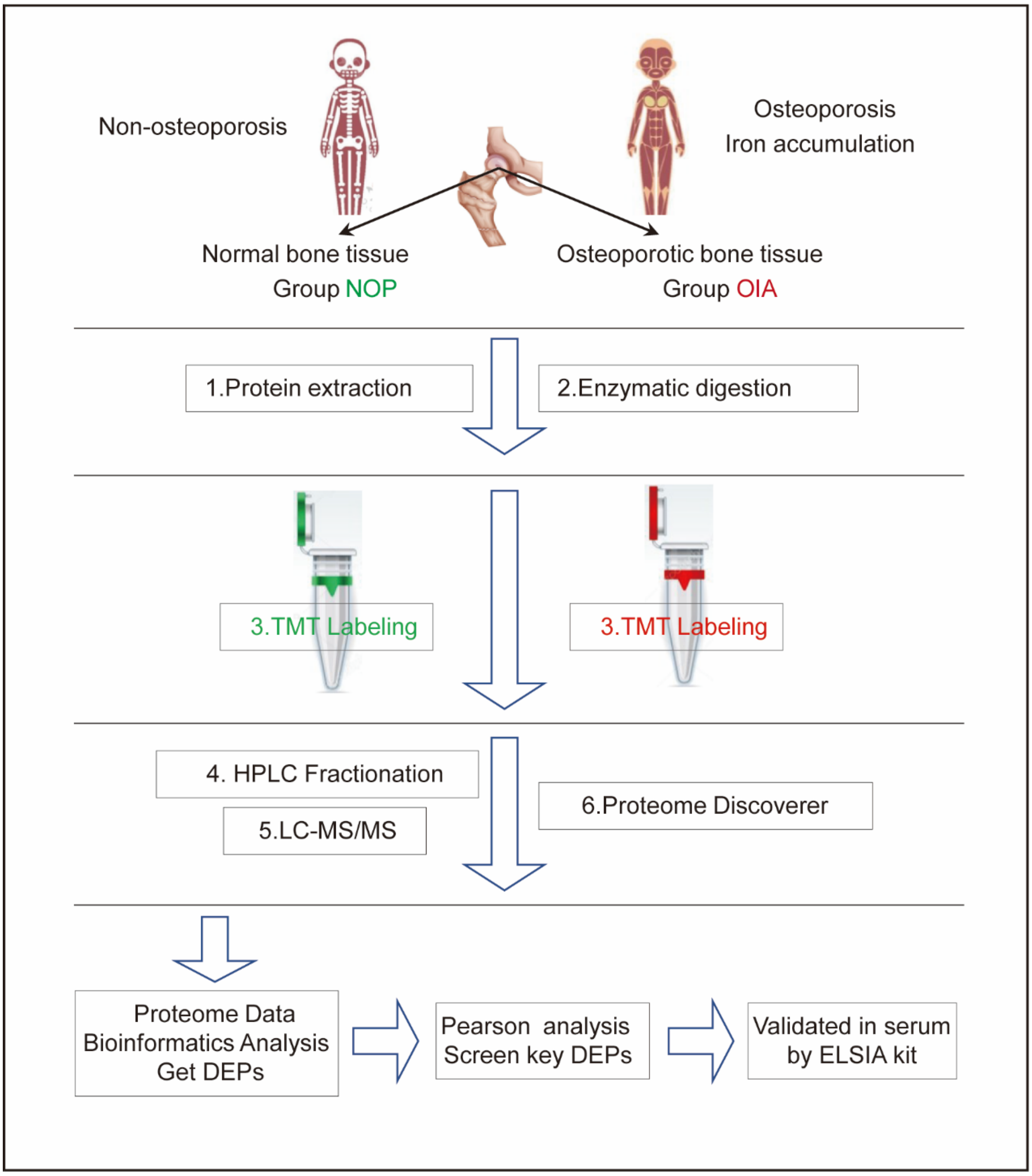

Supplementary Figure 3 | Quantitative proteomics workflow and general analysis of human clinical bone tissues. Workflow for quantitative mass spectrometry profiling. For data acquisition, we grouped the 10 samples into 2 experimental groups for 10-plex LC–MS/MS tandem mass tag (TMT) isobaric labelling. For data processing and quantification, we used TMT-Integrator and generated a combined protein expression matrix for several experiments used for downstream analyses, including hierarchical clustering, differential expression tests, statistical analysis, and biological inference.

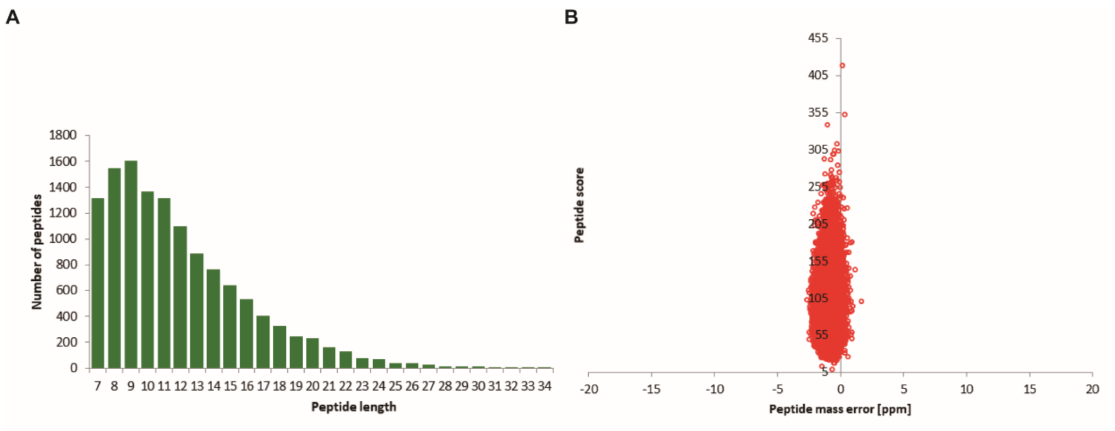

Supplementary Figure 4 | Proteome measurement in human bone tissue meets the quality control requirements. **(A)** Length distribution of all identified peptides. Most of the peptides contain between 7 and 20 amino acids, which is in line with the general rules based on trypsin enzymatic hydrolysis and HCD fragmentation. The distribution of peptide lengths identified by mass spectrometry meets the quality control requirements. **(B)** Mass accuracy distribution of the mass spectrometer. The first-order mass error of most spectra is within 5 ppm, which is in line with the high-precision characteristics of Orbitrap mass spectrometry.

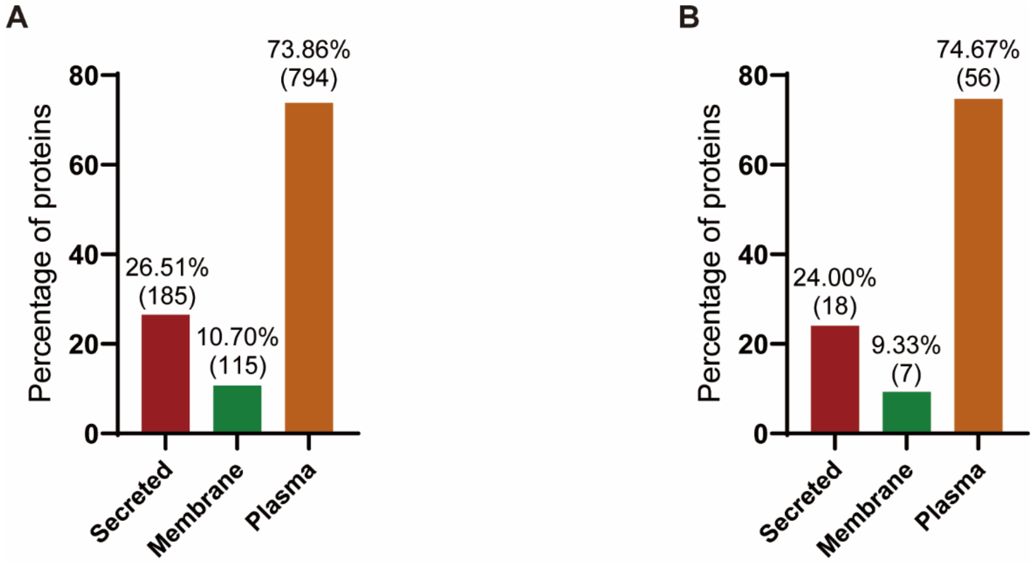

Supplementary Figure 5 | Protein types of non-differentially expressed and DEPs. **(A)** The number and percentage of secreted proteins, membrane proteins and plasma proteins among the no-differentially expressed proteins. **(B)** The number and percentage of secreted proteins, membrane proteins and plasma proteins among the differentially expressed proteins.

Supplementary Table 1 | Number of DEPS contained in Murine OB/OC genes.

	Included DEPs	Percentage of all DEPs
Murine OB genes	66	88%
Murine OC genes	54	72%

Supplementary Table 2 | Person analysis of protein level and bone mass in patients.

		UBA6	PSMD3	TXNL1	CALU	CD5L	SH3BGRL
Hip-T	R	.718*	.363	.471	.784**	.430	.553
score	P Value	.019	.302	.169	.007	.215	.098
		F13B	TPM3	TUBB	PFN1	ELANE	GSTP1
Hip-T	R	-.631	.567	.773**	-.614	-.821**	-.667*
score	P Value	.051	.088	.009	.059	.004	.035
		ITIH2	IBSP	CPN2	GCSH	PSMA2	FKBP2
Hip-T	R	-.561	-.586	.659*	.791**	.649*	.627
score	P Value	.092	.075	.038	.006	.043	.052
		SERPINB10	NDUFV1	HNMT	HSD17B4	COPB1	RAD23A
Hip-T	R	.609	.727*	.759*	-.780**	.862**	.533
score	P Value	.062	.017	.011	.008	.001	.112
		HNRNPU	RPL18	CRYZ	LTBP1	PTGR1	SEPT2
Hip-T	R	.777**	.776**	.770**	-.630	.796**	.714*
score	P Value	.008	.008	.009	.051	.006	.020
		FLOT1	H6PD	SDPR	GOT2	PGK1	F10
Hip-T	R	.663*	.634*	-.705*	.576	-.792**	-.561
score	P Value	.037	.049	.023	.081	.006	.092
		QDPR	TPM1	CNP	CLU	LAMP1	CKB
Hip-T	R	.546	.844**	.807**	-.643*	-.717*	.764*
score	P Value	.102	.002	.005	.045	.020	.010
		ATP5D	CMPK1	YWHAB	SAA4	IGFALS	DLST
Hip-T	R	.789**	.836**	.675*	-.471	-.631	.718*
score	P Value	.007	.003	.032	.169	.051	.019
		ADK	RAB5B	RPS7	CALM1	SNRPD1	RPL11
Hip-T	R	.684*	-.775**	.667*	.747*	-.651*	.724*
score	P Value	.029	.008	.035	.013	.042	.018
		TXNRD1	DCXR	PGM2	COL12A1	TUBB6	SH3BGRL3
Hip-T	R	.699*	.582	.596	.790**	.712*	.765**
score	P Value	.025	.077	.069	.007	.021	.010
		IGHG1	IGHA2	IGLV743	GNAS	TUBA4A	NUCB2
Hip-T	R	.725*	.553	-.521	.681*	-.672*	.698*
score	P Value	.018	.098	.122	.030	.033	.025
		LAMP2	DARS	ORM2	PPA2	NENF	COPS3
Hip-T	R	-.773**	.750*	-.511	.739*	.757*	.658*
score	P Value	.009	.012	.131	.015	.011	.039
		ANP32A	NSF	RPL28			
Hip-T	R	.680*	-.784**	.776**			
score	P Value	.030	.007	.008			

*$p<0.05$, **$p<0.01$

Supplementary Table 3 | Person analysis of protein level and serum ferritin in patients.

		UBA6	PSMD3	TXNL1	CALU	CD5L	SH3BGRL
Fer	R	-.381	-.529	-.449	-.353	-.474	-.417
	P Value	.277	.116	.193	.316	.166	.230
		F13B	TPM3	TUBB	PFN1	ELANE	GSTP1
Fer	R	.327	-.513	-.408	.370	.422	.645*
	P Value	.356	.129	.242	.292	.225	.044
		ITIH2	IBSP	CPN2	GCSH	PSMA2	FKBP2
Fer	R	.363	.418	-.489	-.389	-.537	-.326
	P Value	.303	.230	.152	.266	.110	.358
		SERPINB10	NDUFV1	HNMT	HSD17B4	COPB1	RAD23A
Fer	R	-.462	-.581	-.339	.516	-.638*	-.456
	P Value	.179	.078	.338	.127	.047	.185
		HNRNPU	RPL18	CRYZ	LTBP1	PTGR1	SEPT2
Fer	R	-.583	-.419	-.382	.436	-.445	-.368
	P Value	.077	.228	.276	.207	.197	.296
		FLOT1	H6PD	SDPR	GOT2	PGK1	F10
Fer	R	-.519	-.414	.482	-.338	.595	.429
	P Value	.124	.234	.158	.340	.070	.216
		QDPR	TPM1	CNP	CLU	LAMP1	CKB
Fer	R	-.460	-.567	-.452	.475	.606	-.386
	P Value	.181	.087	.190	.165	.063	.271
		ATP5D	CMPK1	YWHAB	SAA4	IGFALS	DLST
Fer	R	-.554	-.446	-.455	.482	.342	-.378
	P Value	.096	.196	.186	.158	.334	.282
		ADK	RAB5B	RPS7	CALM1	SNRPD1	RPL11
Fer	R	-.407	.690*	-.365	-.603	.603	-.516
	P Value	.243	.027	.299	.065	.065	.127
		TXNRD1	DCXR	PGM2	COL12A1	TUBB6	SH3BGRL3
Fer	R	-.448	-.561	-.383	-.365	-.573	-.537
	P Value	.194	.092	.275	.299	.083	.110
		IGHG1	IGHA2	IGLV743	GNAS	TUBA4A	NUCB2
Fer	R	-.307	-.424	.535	-.416	.336	-.421
	P Value	.388	.222	.111	.231	.342	.226
		LAMP2	DARS	ORM2	PPA2	NENF	COPS3
Fer	R	.648*	-.468	.367	-.451	-.431	-.509
	P Value	.043	.173	.297	.191	.213	.133
		ANP32A	NSF	RPL28			
Fer	R	-.380	.580	-.363			
	P Value	.278	.079	.302			

*$p<0.05$, **$p<0.01$
